# Homophily influences ranking of minorities in social networks

**DOI:** 10.1038/s41598-018-29405-7

**Published:** 2018-07-23

**Authors:** Fariba Karimi, Mathieu Génois, Claudia Wagner, Philipp Singer, Markus Strohmaier

**Affiliations:** 1 GESIS – Leibniz-Institute for the Social Sciences, Cologne, Germany; 2 University of Koblenz-Landau, Koblenz, Germany; 3 RWTH Aachen University, Aachen, Germany

## Abstract

Homophily can put minority groups at a disadvantage by restricting their ability to establish links with a majority group or to access novel information. Here, we show how this phenomenon can influence the ranking of minorities in examples of real-world networks with various levels of heterophily and homophily ranging from sexual contacts, dating contacts, scientific collaborations, and scientific citations. We devise a social network model with tunable homophily and group sizes, and demonstrate how the degree ranking of nodes from the minority group in a network is a function of (i) relative group sizes and (ii) the presence or absence of homophilic behaviour. We provide analytical insights on how the ranking of the minority can be improved to ensure the representativeness of the group and correct for potential biases. Our work presents a foundation for assessing the impact of homophilic and heterophilic behaviour on minorities in social networks.

## Introduction

Social networks are comprised of individuals with a variety of attributes, such as race, age, educational background, or gender. Commonly, these attributes are distributed unequally in the population. For example, in many schools across the United States and Europe, Asian or Black students form a minority^1^; similarly, women are usually a minority in science and engineering^2^. In parallel, homophily, the tendency to associate with similar others, is observed in many social networks, ranging from friendship to marriage to business partnerships^1,3–6^. However, the extent to which homophilic behaviour combined with group size differences has an effect on the structure of a social network and ranking of minorities is not known.

Understanding the factors that impact the ranking of minorities has gained importance in recent years, since algorithms have been more and more widely used for ranking individuals in various application domains, including search engines^7–9^, recommender systems^10,11^, or hiring processes^12–14^. These rankings are critical, since they can impact the influential power of individuals and the opportunities afforded to them. Rankings are commonly based on the topological structure of networks, and hence, the position of individuals in their social network significantly influences their ranks^15–17^. In particular, for networks in which one group of individuals is smaller in size (minority), global ranking can have a crucial impact on the representation of the whole group. A biased algorithm could create situations in which (i) high-ranked minority members become less noticeable globally and therefore less influential in society, (ii) a minority feels ignored or overlooked by the wider public, also known as the invisibility syndrome^18^. It is thus fundamental to understand the effect of group sizes and the different mechanisms of tie formation on the ranking of minorities in social networks.

**Figure 1:**
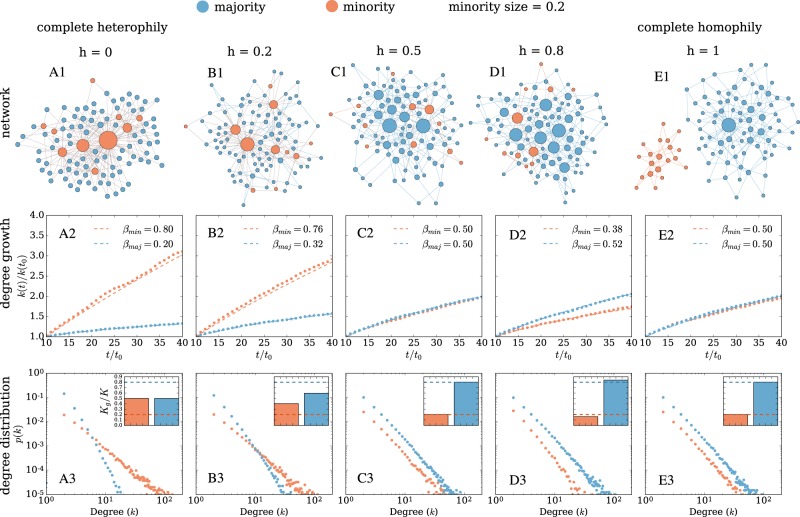
Disproportionate degree ranks and asymmetric effects of homophily on Barabási-Albert networks with minority and majority groups and homophily. The minority group (orange nodes) represents 20% of the population. Homophily is regulated by the parameter *h*. Panel A shows a maximally heterophilic network (*h* = 0). As homophily increases, the likelihood for nodes to connect with other nodes of the same color increases. Panel E finally shows a maximally homophilic network (*h* = 1.0). The top row is a schematic of the network topology generated from the model (Eq. (1)) for a small network with 100 nodes. The second row represents the resulting degree growth during the simulation. In the heterophilic regime (0 ≤ *h* < 0.5), the degree of the minority group grows faster than majority. In the homophilic regime (0.5 < *h* ≤ 1) the growth of the degree slows down for minorities. The third row represents the degree distribution generated by the model for the two types of node. The inset in the third row depicts the share of total degree for minority and majority groups and the dashed lines show the fraction size of the group. For the second and third row the results are given for a network with *N* = 5000 nodes, and averaged over 20 simulations.

In this study, we focus on two main mechanisms for the formation of ties: homophily^3^ and preferential attachment^19^, and systematically study how relative size differences between groups in social networks, with various levels of homophily, impact the ranking of nodes in synthetic and real-world networks. In recent years, models have been proposed that consider homophily^20,21^, or a combination of homophily and preferential attachment in the tie formation process^22–24^. We build on these models by systematically exploring the parameter range for homophily and group size differences and offer analytical and empirical evidence on the emergent properties of networks and the ranking of groups. In our settings, the notion of minority and majority refers to the relative size of the groups in the social network. We define *rank* as the importance of the node in the network and the ability of the node to receive information. Our results (cf. Fig. 1 top row for an illustration) show that the degree rank of nodes in such settings is generally *disproportionate*—i.e. ranking is not proportional to the size of the group and varies with homophily. We find that while minority nodes show higher degree ranks in heterophilic networks, they exhibit lower degree ranks in homophilic networks. Surprisingly, ranking has an *asymmetrical* and non-linear effect in both homophilic and heterophilic regimes. We provide an analytical solution that predicts the exponent of the degree distribution and demonstrates the presence of this asymmetric effect. We finally show evidence of a disproportionate ranking in four empirical networks of sexual contacts, dating contacts, scientific collaboration, and scientific citation with different ranges of homophily and group size.

In the following sections, we first present the analytical and numerical results of the model, showing the effect of homophily and group sizes on the degree ranks of nodes in social networks. We then discuss the impact of the parameters on the ranking of nodes that belong to groups of different sizes. Finally, we demonstrate that our model captures well the properties of real cases, such as the degree distributions and ranks of the majority and minority in empirical social networks with different group sizes and different degrees of homophily.

## Results

### Model

We use the well-known model of preferential attachment proposed by Barabási and Albert^19^, and incorporate homophily as an additional parameter to the model^23,24^. Thus, the mechanism of tie formation in our model is influenced by the interplay between preferential attachment, via the degree of nodes, and homophily, via the node attributes. A variant of this model, known as the fitness model, was first proposed by Bianconi and Barabási^25^. In this model, the probability of a connection is the product of the degree and fitness of the node. However, the fitness of a node is assumed to be constant regardless of the presence of other nodes. In our model, the fitness of a node also depends on the attributes of other nodes.

We model social networks with two types of nodes that are initially labeled as *a* or *b*. In Fig. 1 they are shown with two colors, blue and orange. The label of a node represents the node’s attribute. Next, we define a tunable homophily parameter, *h*, that regulates the tendency of nodes to connect with others based on their attributes. We refer to the nodes of the same attribute as a *group*. The homophily parameter ranges between 0 to 1, *h* ∈ [0, 1], where 0 means that nodes from one group connect only to nodes from the other group (opposing attributes, complete heterophily), 1 means that the nodes connect only with nodes of the same group (similar attributes, complete homophily), and 0.5 indicates a homogeneous mixing with respect to group affiliation. The model consists of *N* nodes and the two attributes are initially assigned to nodes with given sizes. We call *f*_*a*_ the fraction of nodes belonging to group *a*, and *f*_*b*_ = 1 − *f*_*a*_ the fraction of nodes belonging to group *b*. We shall refer to group *a* as the minority and group *b* as the majority, so that *f*_*b*_ ≥ *f*_*a*_. At each time step, a newly arriving node *j* randomly attaches to *m* pre-existing nodes by evaluating their degree and group membership. Multiple linkage between two nodes is not allowed. The probability of node *j* to connect to node *i* is given by:

1 $${{\rm{\Pi }}}_{i}=\frac{{h}_{ij}{k}_{i}}{\sum _{l}{h}_{lj}{k}_{l}}$$

where *k*_*i*_ is the degree of node *i* and *h*_*ij*_ is the homophily between nodes *i* and *j*. Note that we assign the group membership of the nodes and determine the homophily between nodes based on their group membership before creating the networks. In the case of complete homophily, if the arrival node does not encounter any members of the same group, it can remain isolated until it encounters a new node from the same group.

In general, the homophily parameter defines the probability of connection within and between groups. For example, in the case of two groups, we may have two homophily parameters: *h*_*aa*_ (probability of connection between members of group *a*), *h*_*bb*_ (probability of connection between members of group *b*), and the probability between groups (*h*_*ab*_ and *h*_*ba*_), which are the complementary probabilities (*h*_*ab*_ = 1−*h*_*aa*_, *h*_*ba*_ = 1−*h*_*bb*_). As a simplification, one can assume that homophily is regulated by only one parameter *h*, considering that homophily is symmetric and complementary: *h*_*aa*_ = *h*_*bb*_ = *h* and *h*_*ab*_ = *h*_*ba*_ = 1−*h*. In this paper, we first provide the results for the simple case of symmetric homophily and then discuss the asymmetric homophily.

Note that this model generates undirected networks. Since our main focus is modelling social networks, it is realistic to assume that a social link cannot be established if it is not trustful and reciprocal. Online social networks such as LinkedIn, Facebook and Google scholar are based on such a mechanism for link creation. In undirected networks, one important centrality measure, namely, PageRank centrality can be approximated by the degree centrality^26^. There are a few challenges in extending the model to directed graphs. First, many social networks are intrinsically undirected. Second, the model of directed graphs does not take into consideration the degree correlations that can exist in real networks and ultimately can impact node’s ranks^27,28^. Therefore, addressing all these issues are beyond the scope of this paper and we leave them for future work.

### Degree growth

Figure 1 (second row) illustrates the dynamics of the degree growth when tuning homophily. The minority fraction is fixed to 0.2. Our model covers the whole range of network topologies. For 0 ≤ *h* ≤ 0.5 the network is heterophilic, and for 0.5 ≤ *h* ≤ 1 the network is homophilic. In the heterophilic regime, the degrees of the minority nodes grow faster than the degree of the majority. Complete heterophily (*h* = 0) leads to the formation of a bipartite network. The difference in the degree growth reduces gradually as heterophily decreases, until we reach the homogeneous mixing case (*h* = 0.5), in which groups do not matter anymore and we recover the original Barabási-Albert growth model for both groups.

In the homophilic regime (0.5 ≤ *h* ≤ 1), the degrees of the majority nodes grow faster than the degrees of the minority nodes until a certain point *h* ≃ 0.8. After that, the difference in growth decreases until we reach the fully homophilic case (*h* = 1) in which the network is split between the two groups, each having the same degree growth. Such extreme homophilic cases resembles societies in which women and men are completely segregated at schools or some universities, e.g., in Iran or Saudi Arabia^29^.

Figure 1 (third row) illustrates the degree distributions of the groups as a result of their degree growth. The insets show the fraction of the total degree of the group in compare with the relative group sizes (orange and blue dashed lines). In the heterophilic regime, the minority receives more degree than what it is expected from its size and in the homophilic regime is the opposite but asymmetrical.

### Impact of homophily and group size on degree distribution

**Figure 2:**
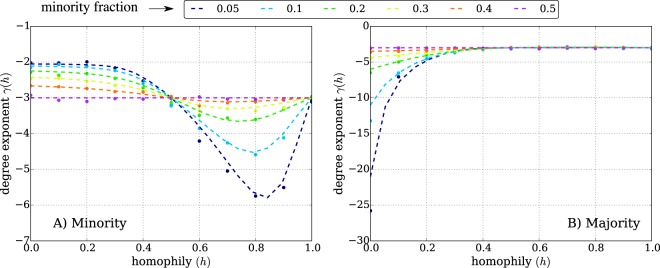
The analytical and numerical exponent of the degree distribution for the minority (**A**) and majority (**B**) as a function of homophily, for various minority sizes. The degree distributions follow a power-law *p*(*k*) ∝ *k*^*γ*^ in which the exponent of the distribution (*γ*) depends on homophily (*h*) and the minority fraction (shown by different colors). The dashed lines are the expected degree exponents given by our analytical derivation (see Methods) and the dots represent the fitted value from the simulations of over 5,000 nodes. The analytical results are in excellent agreement with simulation. The minority fraction ranges from 0.05 to 0.5. For minority nodes (**A**), in the heterophilic regime (*h* < 0.5), the degree exponent ranges from −2 to −3, which shows the advantage these nodes have as their degrees grow to large values. In the homophilic regime (*h* > 0.5), the exponent shows a non-linear behaviour: first the degree exponent decreases, which means that degree growth for the minority nodes becomes limited, and they are thus less well-connected. However, this effect is compensated in high homophilic regime by in-group support, which explains why the exponent increases for *h* > 0.8. For majority nodes (**B**), in the heterophilic situation the growth of their degree is limited, in particular for small minority fractions. In the homophilic regime, the exponent of the majority degree always remains close to −3: the majority nodes do not gain extra advantage due to the size of their group.

Figure 2 shows the evolution of the exponent of the degree distribution for the minority (Fig. 2A) and majority (Fig. 2B) nodes when we tune homophily and group size. We derive analytically the exact exponent of the degree growth and the degree distribution as a function of homophily (*h*) and minority size (*f*_*a*_) (see Methods). The degree exponent illustrates the ability of nodes to stretch their degrees to high values and thus reach higher degree rank. Let us denote the degree distribution *p*(*k*) ~ *k*^*γ*^, where *γ* is the exponent of the degree distribution. When both groups are of equal size (*f*_*a*_ = 0.5), the model recovers the exponent *γ* = −3 for the degree distributions of both groups, as predicted from the classical Barabási-Albert model. In the heterophilic regime (*h* < 0.5), as the size of the minority decreases, the exponent of the degree distribution of the minority increases, which indicates that the degree of the minority nodes reaches larger values. The opposite situation occurs for the majority: as the size of the minority decreases, the exponent of the degree decreases, which indicates that the degree of majority nodes is limited to smaller values.

The homophilic regime (*h* > 0.5) exhibits an interesting behaviour. While the exponent of the degree distribution for the majority does not change much when we tune group size or homophily, there is a non-linear effect for the minority. As homophily increases, the exponent decreases until a certain homophily value (*h* ≃ 0.8), and increases afterwards (see Fig. 2A). In the extreme homophilic case (*h* = 1.0) the degree growth of both groups is similar to the homogeneous mixing case (*h* = 0.5) and so are the exponents of the degree distributions.

This non-linear behaviour can be explained by the interplay between homophily and relative group size differences. Both determine the amount of competition faced by the nodes from different groups. For the majority, heterophilic conditions are not beneficial (in terms of degree exponent), since majority of nodes are mostly attracted to the minority of nodes which makes the minority group extremely popular. Therefore, majority nodes have difficulties competing for the attention of the newly arriving nodes. In the homophilic regime however, the majority is relatively indifferent because they compete for attention mostly among themselves.

For the minority, heterophilic situations are the most beneficial. They receive most of the attention of the majority, and the competition for attention among minority nodes is relatively low since they are a small group. In homophilic situations, however, it is much more difficult for minority nodes to attract newly arriving nodes due to the competition with the majority, which is not only larger in size but also contains more popular nodes. The situation worsens in the middle range of homophily (*h* = 0.8) in which minority nodes are not only in a disadvantage to attract nodes from the majority group but also some nodes from their own group due to fierce competition with the majority. However, in the case of extreme homophily, no competition exists between the nodes of different groups, and thus both groups compete only among themselves. The degree of the nodes in both groups grow similarly, and the degree distributions are the same as in the homogeneously mixed case, with the only difference that the network is now split between the two groups.

Interestingly, the evolution of the degree exponent in the homophilic regime exhibits a parabolic shape. This suggests that our model despite its simplicity displays a non-trivial interplay between homophily and preferential attachment that cannot be explained only by the Barabási and Albert (BA) model. Although it is possible to recover two cases of the model - neutral or complete homophily- the BA model alone cannot explain the behaviour of the degree exponent in other cases of the intermediate range of homophily, in particular, in the homophilic range 0.5 < *h* < 1. In addition, the tipping point around *h* ≃ 0.8 indicates that there can be the same degree exponent for two different values of homophily. That means, considering only aggregated properties of the network such as the degree distribution is not sufficient to determine the microscopic behaviour of the nodes such as tie formation mechanism. Besides, the minimum value in the parabolic curve for minority suggests that the medium range of homophily can be disadvantageous for the degree of the minority to grow.

### Ranking of minorities

So far we have observed that homophily and the difference in group sizes have an effect on the degree growth and the degree distribution of groups. Despite the simplicity of the model, the outcome of social interactions on the ranking of groups is striking.

**Figure 3:**
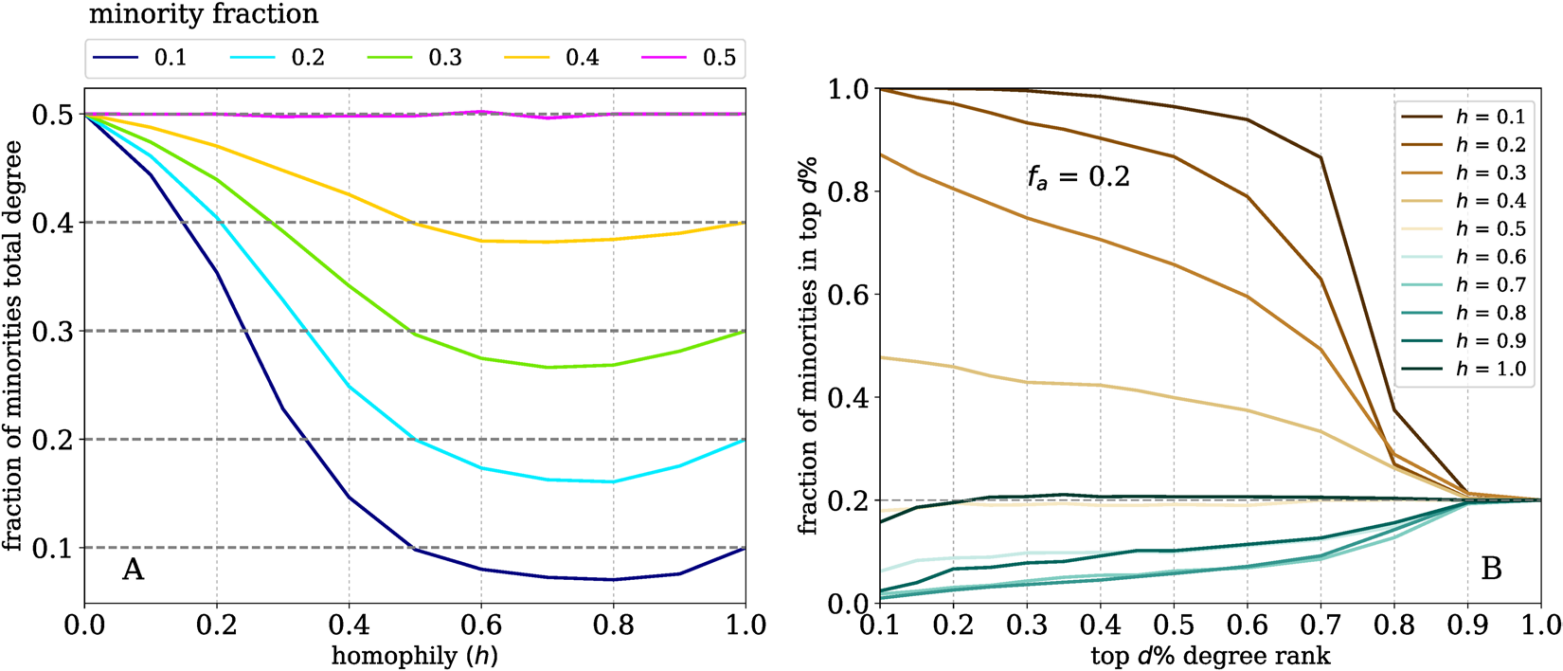
Ranking of minorities as a function of relative group size and homophily. (**A**) Average cumulative degree of the minority as a function of homophily, for different minority fractions (0.1–0.5). In a balanced population (0.5, pink line), both groups share half of the links, independently of the level of homophily. As the size of the minority decreases, the inequality in the share of degree increases. In a homogeneous-mixing case (*h* = 0.5), the rank corresponds to the expected population size shown by the grey dashed lines. In heterophilic regimes (0 ≤ *h* < 0.5), the minority takes advantage of the population size effect. In homophilic regimes (0.5 < *h* ≤ 1), we observe that the degree of minorities is below the expectation and it is recovered only in the extreme homophilic case (*h* = 1) by full in-group support. (**B**) Fraction of minority nodes that are found in the top d% of nodes with the highest degree. The fraction of nodes belonging to the minority (*f*_*a*_) is set to 0.2. If the group membership does not impact the attractiveness of nodes, we expect that the presence of the minority in the top d% is the same as its relative size (dashed line). However, we see that the results are sensitive to homophily. In the heterophilic case (0 ≤ *h* < 0.5), minorities are over-represented in the top d%. In the homophilic case (0.5 < *h* ≤ 1), minorities are under-represented in the top d%. In the case of homogeneous mixing (*h* = 0.5) or complete homophily (*h* = 1.0), minorities are presented in the top d% as expected from their relative size. Note that the results for the majority group is only the complementary of those for the minority.

First, we examine the total degree ranks. Figure 3A depicts the average total degree share of the minority as a function of homophily. Colors represent different minority sizes. The results for the majority group are complementary. In the extreme heterophilic case (*h* = 0), a minority group that represents 20% of the total population (light blue line) receives 50% of the total degree (*i.e*. 50% of the link ends are attached to minority nodes). This result resembles the concept of majority illusion, in which the majority of nodes perceives the opinion of the minority as the majority opinion because they are exposed mainly to minority nodes^30^. As the homophily between groups increases up to 0.5, the average total degree decreases to what we would expect from the size of the minority (dashed grey lines). In the homophilic case (0.5 ≤ *h* ≤ 1), the degree drops below this expectation, and thus the minority group as a whole is penalised. In the extreme homophilic situation (*h* = 1), the minority group can take advantage of full in-group support and the degree returns to the expectation, proportional to the group size.

If we wish to examine only the top-ranked nodes, which is a realistic scenario for users who want to explore a ranked list of items (as in search user interfaces), the results are even more striking. Figure 3B illustrates the probability of finding minorities in the top *d*% of nodes ranked by degree. For example, *d* = 0.2 means the fraction of nodes in the top 20% of the nodes ranked by degree. In the heterophilic case (brown shades), nodes from the minority are over-represented in the top- ranked nodes. In the homophilic case (green shades), nodes from the minority are underrepresented, an effect especially important for small top *d*%. Given the fact that nodes with high degree are very stable in terms of their rank^31^, these results suggest that in homophilic networks, the majority stabilises its position at high ranks and leaves little opportunity for minorities to appear in the top ranks. In heterophilic cases, the roles are reversed: minority nodes stabilise their position at high ranks.

Given the fact that many social networks are homophilic with respect to attributes such as gender or ethnicity, our results suggest that in homophilic networks majorities occupy the high ranks and minorities tend to appear towards the lower ranks compared to what would be a proportionate representation of the population. In a recent Twitter study, the authors found empirical evidence for this effect^32^. They showed that among the top individuals with the highest numbers of followers, white men are over-represented compared to their population size. In addition, the authors showed empirical evidence for the nature of the asymmetric homophily that can exist among groups. However, the paper does not explain what are the underlying social mechanisms that generate this inequality given the homophily and group sizes.

### Access to information

Homophilic or heterophilic interactions not only impact the rank of nodes in terms of degree, but can also impact the ability of nodes to receive information. In the context of scientific collaboration it can be the ability to access novel ideas or awareness of career opportunities. In one study on Twitter, Halberstam and Knight showed that users affiliated with majority political groups, relative to the minority group, have more connections and are exposed to more information. They also found that users are disproportionately exposed to like-minded information and that type of information reaches like-minded users more quickly^33^.

Although a perfect segregation causes the overall network characteristics and rank of the two groups to be similar (complete homophily), a node’s ability to receive information is clearly changing as the two groups become more segregated. To examine the ability of nodes to access information, we model information diffusion as a simple susceptible-informed (SI) process^34^. Initially one random source is chosen as an informed node and all other nodes are susceptible. The probability for adopting the information is fixed. We then propagate the information on the network until all possible transmission routes have been tested. We compute two measures: (i) the fraction of information from a random source successfully reaches a random target; (ii) the average arrival time of the information. In each simulation, the source and the target are chosen at random and we average the results over many iterations. We therefore have four different paths of transmission: from minority to minority (min-min), minority to majority (min-maj), majority to minority (maj-min) and majority to majority (maj-maj).

**Figure 4:**
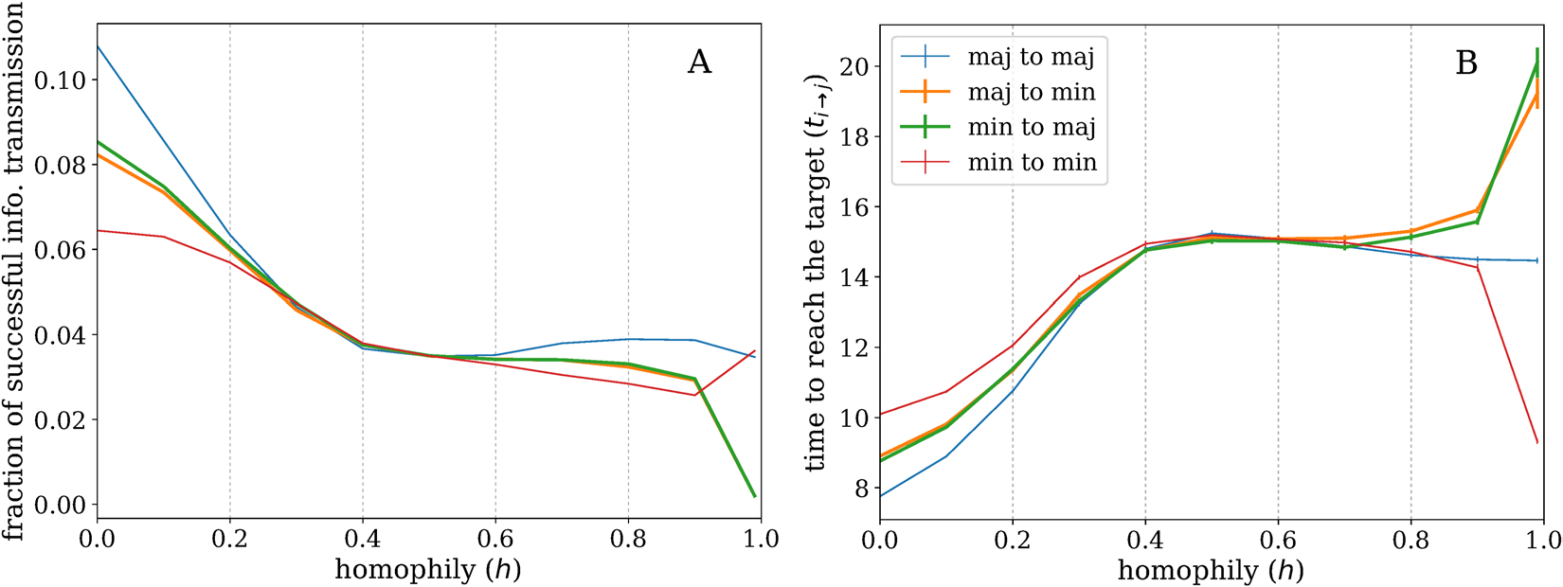
Fraction of successful transmission and average arrival time of information from a random source to a random target for different groups of nodes. (**A**) Fraction of information that successfully reached the target. As homophily increases the information has harder time reaching a majority node from a minority node and vice versa. (**B**) Average arrival time of information. Average arrival time is shorter in heterophilic networks and it increases as the network become homophilic. In the extreme homophilic case, arrival time from majority to minority and vice versa (green and orange lines) increases dramatically until it goes to infinity for complete homophily. The arrival time for minority to minority group decreases in the extreme homophily due to their smaller group size compared to majority. In all cases, *N* = 2000, minority fraction is set to 0.2, the probability of information transmission is set to 0.2 and results are averaged over 2000 simulations.

Figure 4A displays the fraction of successful transmissions from source to target and Fig. 4B the average arrival time of information. We set the minority fraction to 0.2 and the probability of information transmission to 0.2. In the heterophilic regime, information is overall more likely to diffuse faster due to the effect of the minority hubs, which concentrate the links. The difference between the two groups is due to the same split in terms of roles: the majority nodes have an advantage as they are connected to minority hubs, which facilitates the spread of information to other majority nodes. Minority nodes have to rely on majority nodes to transmit the information to other minority nodes, which is less likely to happen due to the low degrees of the majority.

In the homophilic regime, it is harder for information to reach the targets due to the absence of high degree-low degree correlations. More importantly, the arrival time of information from majority to minority and vice versa increases drastically in high homophily (*h* ≥ 0.9) and consequently the probability for information to reach the target decreases. In addition, information is transmitted faster among minority nodes in high homophily due to a smaller number of nodes and thus shorter transmission paths, resembling rumor spreading in a small village. Overall, despite the fact that in highly segregated networks the degree exponent and ranks of groups show similar behaviour, the ability of minority nodes to receive information from majority and vice versa decreases dramatically due to the split of the network.

Note that the SI process in this setting can be thought as a random walk process for undirected graphs. The probability of a random walker starting from a random source to a random target is related to the number of paths that exists between the two nodes. By definition, in undirected graphs, the number of paths are exactly the same in each direction, which explains why the majority-to-minority and minority-to-majority lines overlap.

### Improving the rank

Is there anything can be done to improve the global ranking of a minority? Here, we show two ways in which (i) the minority can overcome the low ranking to improve their visibility (ii) algorithms can be enhanced to enforce a better ranking for the minority.

Individuals belonging to the minority group can improve their ranking in various ways. First and foremost, both the minority and the majority group should strive to increase their heterophilic interactions. That would result in better access to information and better ranking for the minority. Second, the minority group can enhance its general activity in the social network. According to our model, in the general form, each arrival node attaches to *m* pre-existing nodes in the network. By definition, the behaviour of the network in general is not dependent on the choice of *m*. The parameter *m* only determines the lower-bound of the degree and normally it is assumed to be small. Due to the stochasticity of the model and the preferential attachment mechanism, as the network grows, there will be a large heterogeneity in the degree distribution and emergence of hubs as can be observed in the power-law degree distribution. However, one can assume that one group is socially more active than the other group. In this case, we can define two parameters *m*_*a*_ and *m*_*b*_ that regulate the lower-bound of degree for the minority and the majority respectively. We can show analytically that the minority nodes can improve their ranking by increasing their degree activity *m*_*a*_ (see supplementary). In other words, more social engagements can help the minority group to improve its overall visibility and rank. However, in many social networks, it is often difficult if not impossible to change the micro-behaviour of the individuals.

A second way is for ranking algorithms to ensure that the percentage of a minority in top *d*% is reflecting the true fraction of the minority, if not better. This refers to Fig. 3B. If we assume that a network is driven by homophily and preferential attachment, we can analytically determine how much compensation is needed to increase or decrease the ranks (see supplementary).

For example, let us assume a network with symmetric homophily of *h* = 0.8 and a minority fraction of 0.2. Let us also assume that each arrival node initially connects to 10 existing nodes, thus *m* = 10. The exponents of the degree distributions can be then calculated as explained in the Methods. If we wish to consider only top 10% of the nodes, that means to choose nodes that have degree *K* = 20 or higher according to Eq. 15 in the supplementary. In this case, only 8% of the minority nodes will be chosen which is lower than what we would expect from their group size. To correct for that, the algorithm can be adjusted to lower the threshold of the degree for the minority to *K* = 14 and increase the degree threshold for the majority to *K* = 22.

### Ranking of minorities in empirical social networks

We provide evidence for the presence of inequality in degree rankings in real social networks, using four empirical social networks that exhibit various values of group size and homophily: sexual contacts, dating network, scientific collaborations and scientific citations.

We first have to determine the value of homophily in the empirical networks. Established methods to quantify homophily include Newman’s assortativity mixing^35^ and dyadicity^36^. These measures are used to quantify the significance level of outgroup links compared to random expectation. However, real social networks do not necessarily exhibit symmetric homophily. Observing only the edges between groups does not capture this potential asymmetric behaviour between groups. For example, if homophily within the minority (fraction size = 0.2) is 0.1 and the homophily within the majority is 0.7, assortativity mixing according to the Eq. 3 of the ref.^35^ will be close to zero; this is similar to the case in which homophily is equal to 0.5 for both groups. However, in the former case we would expect that the number of edges that exist within the majority group is far greater than the number of edges within the minority group, after correcting for the group size. Therefore, to fully grasp asymmetric homophilic behavior, we need to consider the fraction of links that run between groups. Given the number of links that run between each group and the relation between group sizes, homophily, and degree exponent, we can analytically determine the homophily parameter for each group (see Methods).

The analytical derivation enables us to accurately estimate the value of the homophily parameter in empirical networks by using only the number of edges within each group given the group sizes. We then focus on four examples exhibiting extreme heterophily (sexual contacts, Brazil), high heterophily (dating network PussOKram, POK), moderate homophily (scientific collaborations, DBLP) and high homophily (scientific citations, APS). We assume that all networks are undirected and we focus on one node attribute (e.g. gender or scientific field). The characteristics of the four empirical networks are summarised in Table 1. For detailed description of the networks see the supplementary.

**Table 1:** Characteristics of the empirical networks.

Data	Nodes	Minority	Majority	Homophily (minority, majority)
Brazil	16,730	sex-workers 6,624 (40%)	sex-buyers 10,106	0, 0
POK	29,341	minority 12,868 (44%)	majority 16,473	0.2, 0.17
DBLP	280,200	female 63,356 (22%)	male 216,844	0.57, 0.56
APS	1,853	CSM 695 (37%)	QSM 1,158	0.8, 1.0

The table shows four real-world networks with two groups. Number of nodes, group sizes and homophily values are reported. We report the homophily values for each group (*h*_*aa*_ homophily between minority to minority and *h*_*bb*_ homophily between majority to majority).

To evaluate our model against the data, we compare the exponent of the empirical degree distribution with the exponent generated by our model given the same empirical homophily and group size values. Our model proves to be able to reproduce similar degree exponents as in the empirical networks. The results of the fit can be found in the supplementary.

**Figure 5:**
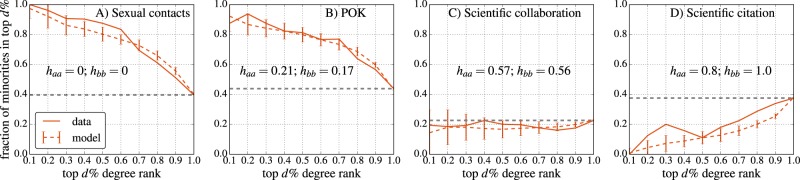
Ranking of minority groups in the top d% degree rank for four empirical networks. (**A**) Sexual contact network (minority = sex workers). (**B**) Online dating network PussOKram (POK). (**C**) Scientific collaboration network (minority = women). (**D**) Scientific citation network (minority = Classical Statistical mechanics (CSM)). The solid orange line is measured on the empirical network and the dashed orange line is the predicted trend, computed using synthetic networks with similar homophily parameters, for 5,000 nodes and averaged over 100 simulations. The dashed gray line is the relative size of the minority, and thus the expected fraction of minority nodes. In the heterophilic cases (**A**,**B**), the minority is over-represented with respect to its size. In the collaboration network where homophily is moderate (**C**), the minority is underrepresented but close to its relative size. In the case of the citation network which is extremely homophilic (**D**), the minority is highly underrepresented. These results provide empirical evidence for a ranking bias in empirical networks.

Furthermore, we examine the top nodes ranked by degree. Figure 5 illustrates the probability of finding minority nodes in the top *d* % of nodes ranked by degree. In the heterophilic cases (sexual contacts and dating networks) Fig. 5A,B, the minority is over-represented with respect to its size. In the scientific collaborations Fig. 5C in which homophily is moderate, the minority rank is close to its expected value. In the case of the scientific citations Fig. 5D which is extremely homophilic, the representation of the minority is highly underestimated. We provide the results of the ranks in synthetic networks with similar homophilic parameters (dashed orange lines). Despite the simplicity of the model, the majority of ranks fall well within the standard deviation of the model. These results provide empirical evidence for a ranking bias in empirical networks and the usefulness of the model to capture and understand such biases.

## Discussion

We have demonstrated analytically and numerically that the degree ranking of nodes is influenced by the relative group size difference and by homophily, and that this influence has an asymmetric and non-linear property. As the size of a minority group decreases, minority nodes benefit more from heterophilic interactions and suffer from homophilic interactions. Minority nodes experience lower rankings mostly in the intermediate range of homophily, in which their ability to attract nodes from both groups is minimum. Minority nodes recover higher degrees by full in-group support when homophily further increases, but they will then suffer from poor accessibility when it comes to receiving information spread by the majority. Although our model makes simple assumptions, such as all members of the same group behaving similarly and being equally active, it lays a theoretical foundation for studying how inherent properties of networks can lead to biases in the ranking of nodes, in particular nodes that belong to minorities. Neglecting the relative group size differences and homophily thus leads to sever consequences for minorities, particularly in search engines or ranking algorithms. As counter measures, we have shown that an increase in the connectivity of minority nodes can mitigate the segregation effect. Another way is for algorithms to be devised to harness relative group size differences and homophily to ensure the representativeness of minorities and correct for potential biases. We have shown how to measure the intensity of the homophilic behaviour in a network, and, knowing this parameter, how much compensation is thus needed.

Our work can be extended in multiple ways. First, it is important to collect more large-scale datasets that represent minority groups. This is, in particular, challenging since sampling methods from networks can potentially impose a bias on the representativeness of minorities^37^, or often hard-to-reach populations are absent from datasets^38^. Second, the model can be extended to account for directionality and multiple attributes in networks or multiplex networks. Third, this model can be used to study community detections in annotated networks^39^ or evaluating the performance of classifiers in machine learning tasks^40,41^. We anticipate that this work will inspire more empirical and theoretical exploration on the impact of network structure on the visibility and ranking of minorities to help establish more equality and fairness in society.

## Methods

Here, we provide the analytical derivation of the degree growth and the exponent of the degree distribution of the model. We do this using two approaches; exact derivation and continuum approximation (see Appendix).

### Exact degree dynamics

Let *K*_*a*_(*t*) and *K*_*b*_(*t*) be the sum of the degrees of nodes from group *a* and *b* respectively. Since the overall growth of the network follows a Barabási-Albert process, the evolution of these quantities verify:

2 $${K}_{a}(t)+{K}_{b}(t)=K(t)=2mt$$

where *m* is the number of new links in the network at each time step *t*. Let us denote the relative fraction of group size for each group as *f*_*a*_ and *f*_*b*_. Considering the general case of asymmetric homophily, the evolution of *K*_*a*_ and *K*_*b*_ is given in discrete time by:

3 $$\{\begin{array}{c}{K}_{a}(t+{\rm{\Delta }}t)={K}_{a}(t)+m({f}_{a}(1+\frac{{h}_{aa}{K}_{a}(t)}{{h}_{aa}{K}_{a}(t)+{h}_{ab}{K}_{b}(t)})+{f}_{b}\frac{{h}_{ba}{K}_{a}(t)}{{h}_{bb}{K}_{b}(t)+{h}_{ba}{K}_{a}(t)}){\rm{\Delta }}t\\ {K}_{b}(t+{\rm{\Delta }}t)={K}_{b}(t)+m({f}_{b}(1+\frac{{h}_{bb}{K}_{b}(t)}{{h}_{bb}{K}_{b}(t)+{h}_{ba}{K}_{a}(t)})+{f}_{a}\frac{{h}_{ab}{K}_{b}(t)}{{h}_{aa}{K}_{a}(t)+{h}_{ab}{K}_{b}(t)}){\rm{\Delta }}t\end{array}$$

which in the limit Δ*t* → 0 gives:

4 $$\{\begin{array}{c}\frac{d{K}_{a}}{dt}=m({f}_{a}(1+\frac{{h}_{aa}{K}_{a}(t)}{{h}_{aa}{K}_{a}(t)+{h}_{ab}{K}_{b}(t)})+{f}_{b}\frac{{h}_{ba}{K}_{a}(t)}{{h}_{bb}{K}_{b}(t)+{h}_{ba}{K}_{a}(t)})\\ \frac{d{K}_{b}}{dt}=m({f}_{b}(1+\frac{{h}_{bb}{K}_{b}(t)}{{h}_{bb}{K}_{b}(t)+{h}_{ba}{K}_{a}(t)})+{f}_{a}\frac{{h}_{ab}{K}_{b}(t)}{{h}_{aa}{K}_{a}(t)+{h}_{ab}{K}_{b}(t)})\end{array}$$

These equations verify that for *h*_*aa*_ = *h*_*bb*_ = 0 and *h*_*ab*_ = *h*_*ba*_ = 1 (perfectly heterophilic network) we get:

5 $$\{\begin{array}{c}\frac{d{K}_{a}}{dt}=m\\ \frac{d{K}_{b}}{dt}=m\end{array}$$

and thus for the evolution of the degree of a single node:

6 $$\{\begin{array}{c}\frac{d{k}_{a}}{dt}=m{f}_{b}\frac{{k}_{a}}{\sum _{i}{k}_{i}}=m{f}_{b}\frac{{k}_{a}}{{K}_{b}(t)}={f}_{b}\frac{{k}_{a}}{t}\\ \frac{d{k}_{b}}{dt}=m{f}_{a}\frac{{k}_{b}}{\sum _{i}{k}_{i}}=m{f}_{a}\frac{{k}_{b}}{{K}_{a}(t)}={f}_{a}\frac{{k}_{b}}{t}\end{array}$$

which gives:

7 $$\{\begin{array}{c}{k}_{a}\propto {t}^{{f}_{b}}\\ {k}_{b}\propto {t}^{{f}_{a}}\end{array}$$

Similarly, for *h*_*aa*_ = *h*_*bb*_ = 1 and *h*_*ab*_ = *h*_*ba*_ = 0 (perfectly homophilic network) we get:

8 $$\{\begin{array}{c}\frac{d{K}_{a}}{dt}=2m{f}_{a}\\ \frac{d{K}_{b}}{dt}=2m{f}_{b}\end{array}$$

and thus for the evolution of the degree of a single node:

9 $$\{\begin{array}{c}\frac{d{k}_{a}}{dt}=m{f}_{a}\frac{{k}_{a}}{\sum _{i}{q}_{i}{k}_{i}}=m{f}_{a}\frac{{k}_{a}}{{K}_{a}(t)}=\frac{{k}_{a}}{2t}\\ \frac{d{k}_{b}}{dt}=m{f}_{b}\frac{{k}_{b}}{\sum _{i}{q}_{i}{k}_{i}}=m{f}_{b}\frac{{k}_{b}}{{K}_{b}(t)}=\frac{{k}_{b}}{2t}\end{array}$$

which gives:

10 $$\{\begin{array}{c}{k}_{a}\propto {t}^{\mathrm{1/2}}\\ {k}_{b}\propto {t}^{\mathrm{1/2}}\end{array}$$

Let us make the hypothesis that *K*_*a*_(*t*) and *K*_*b*_(*t*) are linear functions of time, so that *K*_*a*_(*t*) = *Cmt* and *K*_*b*_(*t*) = (2 − *C*)*mt* given Eq. (2). In the case of two groups, we can simplify the notations by denoting *f*_*a*_ = *f* and *f*_*b*_ = 1 − *f*. Using Eq. (4), we thus have:

11 $$\frac{d{K}_{a}}{dt}=Cm=m(f(1+\frac{{h}_{aa}Cmt}{{h}_{aa}Cmt+{h}_{ab}\mathrm{(2}mt-Cmt)})+\mathrm{(1}-f)\frac{{h}_{ba}Cmt}{{h}_{bb}\mathrm{(2}mt-Cmt)+{h}_{ba}Cmt})$$

and thus:

12 $$C=f(1+\frac{{h}_{aa}C}{{h}_{aa}C+{h}_{ab}\mathrm{(2}-C)})+\mathrm{(1}-f)\frac{{h}_{ba}C}{{h}_{bb}\mathrm{(2}-C)+{h}_{ba}C}$$

All other parameters being known, this equation for *C* can be numerically solved. Within the ranges of values of the parameters, it has three real solutions, but only one in the interval [0, 2] and thus valid in this case. We can then derive the evolution of the degree of a single node for both groups in the general case. Let’s define:

13 $$\begin{array}{rcl}{Y}_{a}(t) & = & {h}_{aa}{K}_{a}(t)+{h}_{ab}{K}_{b}(t)\\  & = & {h}_{aa}Cmt+{h}_{ab}\mathrm{(2}-C)mt\\  & = & mt({h}_{aa}C+{h}_{ab}\mathrm{(2}-C))\end{array}$$

and

14 $$\begin{array}{rcl}{Y}_{b}(t) & = & {h}_{ba}{K}_{a}(t)+{h}_{bb}{K}_{b}(t)\\  & = & {h}_{ba}Cmt+{h}_{bb}\mathrm{(2}-C)mt\\  & = & mt({h}_{ba}C+{h}_{bb}\mathrm{(2}-C))\end{array}$$

For group *a*, we have:

15 $$\begin{array}{rcl}\frac{d{k}_{a}}{dt} & = & m{f}_{a}\frac{{h}_{aa}{k}_{a}}{{Y}_{a}}+m{f}_{b}\frac{{h}_{ba}{k}_{a}}{{Y}_{b}}\\  & = & \frac{{k}_{a}}{t}(\frac{{f}_{a}{h}_{aa}}{{h}_{aa}C+{h}_{ab}\mathrm{(2}-C)}+\frac{{f}_{b}{h}_{ba}}{{h}_{ba}C+{h}_{bb}\mathrm{(2}-C)})\\  & = & \frac{{k}_{a}}{t}{\beta }_{a}\end{array}$$

and thus:

16 $${k}_{a}(t)\propto {t}^{{\beta }_{a}}$$

Similarly, for group *b* we have:

17 $$\begin{array}{rcl}\frac{d{k}_{b}}{dt} & = & m{f}_{b}\frac{{h}_{bb}{k}_{b}}{{Y}_{b}}+m{f}_{a}\frac{{h}_{ab}{k}_{b}}{{Y}_{a}}\\  & = & \frac{{k}_{b}}{t}(\frac{{f}_{b}{h}_{bb}}{{h}_{ba}C+{h}_{bb}\mathrm{(2}-C)}+\frac{{f}_{a}{h}_{ab}}{{h}_{aa}C+{h}_{ab}\mathrm{(2}-C)})\\  & = & \frac{{k}_{b}}{t}{\beta }_{b}\end{array}$$

and thus:

18 $${k}_{b}(t)\propto {t}^{{\beta }_{b}}$$

**Figure 6:**
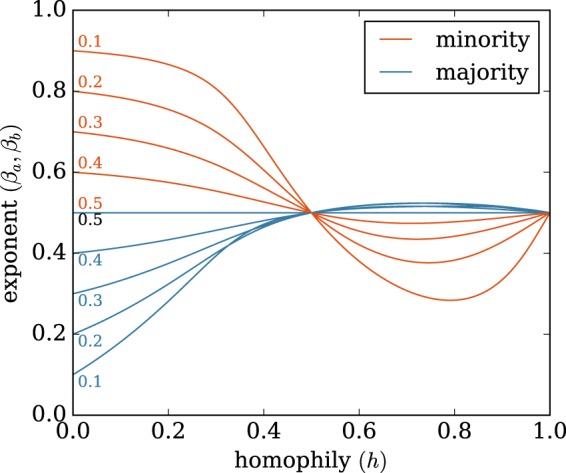
Evolution of the exponents for the degree growth, symmetric homophily. The exponents *β*_*a*_ (minority) and *β*_*b*_ (majority) are defined in Eqs (15 and 17). *h* = *h*_*aa*_ = *h*_*bb*_ is the homophily parameter and the numbers on the curves indicate the fraction of nodes belonging to the minority group (parameter *f*_*a*_).

**Figure 7:**
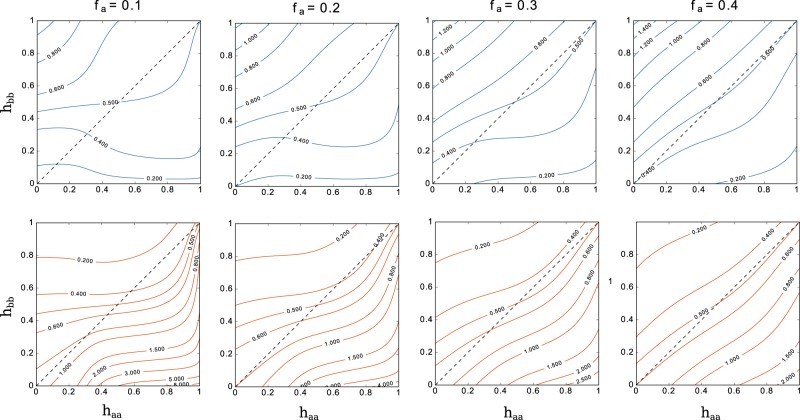
Evolution of the exponents for the degree growth, asymmetric homophily. The exponents *β*_*a*_ and *β*_*b*_ are defined in Eqs (15 and 17). *h*_*aa*_ and *h*_*bb*_ are the homophily parameters. Bottom row shows the behaviour of *β*_*a*_ and top row the behaviour of *β*_*b*_. Columns are ordered according to the fraction of nodes belonging to the minority group (parameter *f*_*a*_), respectively *f*_*a*_ = 0.1, 0.2, 0.3 and 0.4 from left to right. The dashed lines indicate the symmetrical case plotted in Fig. 6.

We plot the evolution of these exponents *β*_*a*_ and *β*_*b*_ in the special case where *h*_*aa*_ = *h*_*bb*_ = *h* and *h*_*ab*_ = *h*_*ba*_ = 1−*h* (Fig. 6). The general case where homophily is not symmetrical is shown in the contour plot in Fig. 7. The dashed lines indicate the previous case of symmetric homophily.

Finally, as has been shown before, there is an inverse relation between the exponent of the degree growth and the exponent of the degree distribution (*p*(*k*) ∝ *k*^*γ*^), as follow^19,25^:

19 $$\gamma =-\,(\frac{1}{\beta }+1)$$

In the case where homophily is equal to 0.5 for both groups, we have *β*_*a*_ = *β*_*b*_ = 0.5, in which the model converges to classic BA model with degree exponent *p*(*k*) ∝ *k*^−3^.

### Estimating asymmetric homophily parameters

The analytical derivations in the previous section enable us to estimate the homophily parameter given the fraction of edges that exist within each group in empirical networks.

In a network with *M* edges, let us call *M*_*aa*_ the number of edges linking two nodes of the group *a* (in-group links), and similarly *M*_*bb*_ the number of edges linking nodes of the group *b*. The probability to have an in-group link in group *a*, which can then be defined as ${m}_{aa}=\frac{{M}_{aa}}{M}$, is given by (see derivation in supplementary):

20 $${m}_{aa}=\frac{{f}_{a}^{2}{h}_{aa}\mathrm{(1}-{\beta }_{b})}{{f}_{a}{h}_{aa}\mathrm{(1}-{\beta }_{b})+{f}_{b}{h}_{ab}\mathrm{(1}-{\beta }_{a})}$$

Similar, we have for the group *b*:

21 $${m}_{bb}=\frac{{f}_{b}^{2}{h}_{bb}\mathrm{(1}-{\beta }_{a})}{{f}_{b}{h}_{bb}\mathrm{(1}-{\beta }_{a})+{f}_{a}{h}_{ba}\mathrm{(1}-{\beta }_{b})}$$

Note that in the general case homophily can be asymmetric, *h*_*ab*_ ≠ *h*_*ba*_. From our previous analytical calculations, we know the relation between the exponent *β*, the group size and the homophily parameter by numerically solving Eq. (12) given *m*_*aa*_ and *m*_*bb*_. We can then solve these nonlinear dynamical equations and determine the expected homophily *h*_*aa*_ and *h*_*bb*_ for group *a* and *b*.

## Electronic supplementary material

Supplementary Information
