# Supplementary material for: Homophily influences ranking of minorities in social networks
Source: Sci Rep. 2018 Jul 23;8:11077. doi: 10.1038/s41598-018-29405-7 (PMC6056555; doi:10.1038/s41598-018-29405-7)
Supplement: Supplementary file 1 — Supplementary Information [file 41598_2018_29405_MOESM1_ESM.pdf]

Homophily influences ranking of minorities in social networks

Fariba Karimi,[1,2] Mathieu Génois,[1] Claudia Wagner,[1,2] Philipp Singer,[1,2] and Markus Strohmaier[1,3]
[1] *GESIS – Leibniz-Institute for the Social Sciences, Cologne, Germany*
[2] *University of Koblenz-Landau, Koblenz, Germany*
[3] *HumTec Institute, RWTH Aachen University, Aachen, Germany*

I. EMPIRICAL NETWORKS

The Brazil network captures sexual contacts between sex workers and sex buyers [10]. The network consists of 16,730 nodes and 39,044 edges. There are 6,624 sex workers and 10,106 sex buyers (minority fraction $f_a = 0.4$). In this network, there exists no edge between members of the same type (the network is bipartite) and consequently the homophily parameter is equal to 0 for both groups, $h_{aa} = h_{bb} = 0$.

The POK network is from an online Swedish dating network called PussOKram.com (POK for brevity) [5] with high heterophily. The data contains 29,341 nodes and 115,684 social contacts. The data initially does not contain node labels. Given the high bipartivity of this network we are able to infer the label of the nodes using max-cut greedy algorithm and results are in agreement with the bipartivity reported in [6]. The assignment of the label is arbitrary and we choose to label the minority group as “minority” and the majority as “majority”. The fraction of minority in this network is 0.43 and the homophily parameters are $h_{aa} = 0.21$ for the minority and $h_{bb} = 0.17$ for the majority.

The DBLP network, which exhibits moderate homophily and relative group size difference, depicts scientific collaborations in computer science extracted from DBLP [1]. To infer the gender of the scientists with high accuracy, we have used name and family name of the scholars in google image to retrieve the first 5 images of each scholar and use Face++ algorithm to infer the gender [7, 8]. We focus on a 4-years snapshot of the network. After removing ambiguous names, the resulting network consists of 280,200 scientists and 750,601 edges (paper co-authorships) with 63,356 female scientists and 216,844 male scientists ($f_a = 0.23$). We measure the homophily among women ($h_{aa} = 0.56$) and among men ($h_{bb} = 0.58$) which indicates slight homophily for both gender.

The APS network captures scientific citations in the American Physical Society corpus that exhibits strong homophily. Citation networks reveal how much attention communities around different topics attribute to each other. We use PACS identifier to select papers on the same topics. In this case we chose statistical physics, thermodynamics and nonlinear dynamical system sub-fields (PACS = 05). Within a specific sub-field there are many sub-topics that form communities of various sizes. To make the data comparable with our model, we choose two sub-topics that are relevant, namely classical statistical mechanics (CSM - 05.20.-y) and quantum statistical mechanics (QSM - 05.30.-d). According to PACS classification in physics, there are two sub-branches in statistical mechanics namely classical statistical mechanics and quantum statistical mechanics. These two branches are highly related since both systems consist of tools to calculate the probability parameters that are determined by a given distribution function. So in principle, both systems solving the same kinds of problems and they only differ based on the type of the distribution function dependent on the nature of the system. The resulting network consist of 1,853 scientific papers and 3,627 citation links. The minority group in these two sub-topics is CSM ($f_a = 0.38$). We find weaker homophily for the CSM papers ($h_{aa} = 0.8$) than for QSM papers ($h_{bb} = 1$), which indicates asymmetric, homophilic behaviour in the citation network.

II. DEGREE EXPONENT IN EMPIRICAL NETWORKS

To evaluate our model against the data, we compare the exponent of the empirical degree distribution with the exponent generated from our model given the same empirical homophily and group size values. To estimate the exponent of the empirical degree distribution we use the maximum-likelihood fitting method [3, 4]. The exponent of the degree distribution generated from the model has been calculated analytically (see Methods). Figure 1 displays degree distribution of minorities and majorities in four empirical networks. The exponent of the degree distributions of networks generated with our model may not have a perfect fit in all cases, but it can explain the trend, and the right-skewed nature of the degree [9]. In fact, the degree fit deviation from the model has been observed in many prominent models of networks [4, 11].

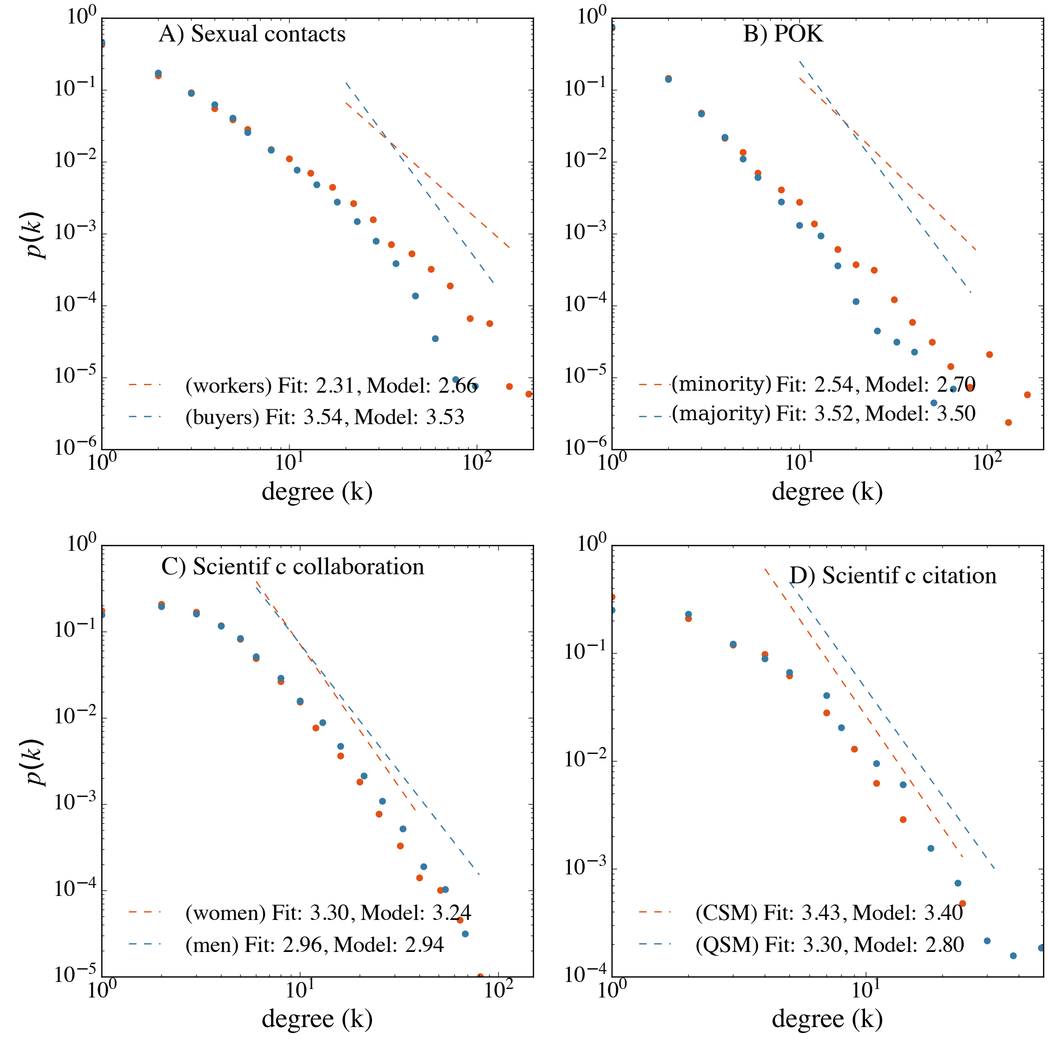

FIG. 1: **Degree distributions for the majority and minority groups in four empirical networks.** A) Sexual contact network with sex-workers (blue) and sex-buyers (orange). B) Online Swedish dating (POK). C) Collaboration network with men (blue) and women (orange). D) American Physical Society (APS) citation network for two topics: Classical Statistical Mechanics (CSM, orange) and Quantum Statistical Mechanics (QSM, blue). The dashed line is the fit using a maximum likelihood estimate. The exponent of the fit (Fit) is compared with the analytical exponent derived from our model (Model). Our model generates a realistic degree exponent for empirical networks with various types of homophily and group sizes.

III. DERIVATION OF THE PROBABILITY OF HAVING AN INTERNAL LINK

We focus on the case of links internal to the group a. The case of the group b will be the exact symmetric.

Let m_{aa} be the probability to establish a link between two nodes of the group a at each arriving node. By construction, it is also the probability to find a link within the group a. Given Eq. (4) of the main text, the probability to generate a link between two nodes of the group a is given by:

$$m_{aa}(t) = f_a \frac{h_{aa} K_a(t)}{h_{aa} K_a(t) + h_{ab} K_b(t)} \tag{1}$$

Given the results of the derivation of the exponents for the degree distributions, we also have:

$$\begin{cases} K_a(t) = Cmt \\ K_b(t) = (2 - C)mt \end{cases} \tag{2}$$

It follows that this probability is a constant and given by:

$$m_{aa} = f_a \frac{h_{aa}C}{h_{aa}C + h_{ab}(2-C)} \tag{3}$$

Given the expression of C in Eq. (12) of the main text, and the expression of β_a given by Eq. (15) of the main text, one can write that:

$$C = f_a + C\beta_a \tag{4}$$

and thus:

$$C = \frac{f_a}{1-\beta_a} \tag{5}$$

Similarly from C and $K_a(t)$, one can express $(2-C)$ starting from the differential equation for $K_b(t)$ (Eq. (4) of the main text):

$$2 - C = f_b \left(1 + \frac{h_{bb}(2-C)}{h_{ba}C + h_{bb}(2-C)}\right) + f_a \frac{h_{ab}(2-C)}{h_{aa}C + h_{ab}(2-C)} \tag{6}$$

Using the expression for β_b (Eq. (17) of the main text), one thus has:

$$2 - C = \frac{f_b}{1-\beta_b} \tag{7}$$

Finally, we find that m_{aa} is given by:

$$m_{aa} = \frac{f_a^2 h_{aa}(1-\beta_b)}{f_a h_{aa}(1-\beta_b) + f_b h_{ab}(1-\beta_a)} \tag{8}$$

and m_{bb} is given by:

$$m_{bb} = \frac{f_b^2 h_{bb}(1-\beta_a)}{f_b h_{bb}(1-\beta_a) + f_a h_{ba}(1-\beta_b)} \tag{9}$$

We verify that these expression give:

- for $h_{aa} = h_{bb} = 1$ (perfect homophily): $m_{aa} = f_a$ and $m_{bb} = f_b$;
- for $h_{aa} = h_{bb} = 0$ (perfect heterophily): $m_{aa} = m_{bb} = 0$;
- for $h_{aa} = h_{bb} = 0.5$ (perfect mixing): in this case we also have $\beta_a = \beta_b = 0.5$ and then $m_{aa} = f_a^2$, $m_{bb} = f_b^2$, and as a consequence $m_{ab} = 2f_a f_b$.

Figure 2 shows the agreement between numerical and analytical results. For simplicity, we fix the value of the homophily parameter in one group and show the relation between tunable homophily and the fraction of edges for the other group. The dashed lines corresponds to the results of the analytical derivation. The value of the homophily parameter extracted from the simulations is shown by the dots.

As the figure shows, in the case of homophily fixed for one group at 0.5 and same group size (panel left), we observe as expected a sigmoid function for both groups. For the largest value of homophily ($h_{aa}, h_{bb} = 1$), the fraction of edges between nodes of the same group converges to the size of the group. As the size of the minority decreases, the gap between the fraction of edges for the minority (orange lines when the majority homophily is fixed ($h_{bb} = 0.5$)) and the majority (blue lines when the minority homophily is fixed ($h_{aa} = 0.5$)) widen. By tuning the group size and fixing the homophily parameter for minorities, the majority gains an advantage by receiving links within itself partly because of the increase in their degree exponent and large group size differences (blue lines).

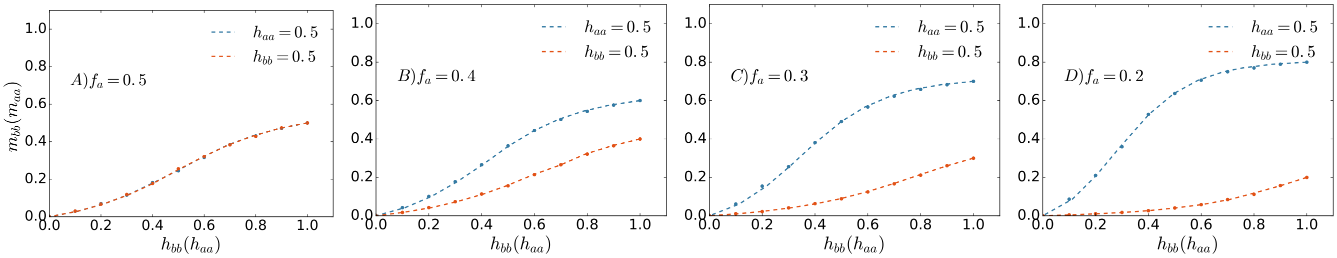

FIG. 2: **Analytical and numerical estimation of the fraction of edges within each group of nodes versus homophily.** Fractions of edges within each group are denoted by m_{aa} and m_{bb}. The homophily parameter is tuned for one group and fixed for another group. Panels from left to right are generated for various minority sizes. The numerical results are shown by points in the plot. The analytical results are shown by dashed lines. As the size of the minority decreases, the gap between the fraction of edges for minority (orange lines when majority homophily is fixed ($h_{bb} = 0.5$)) and majority (blue lines when minority homophily is fixed ($h_{aa} = 0.5$)) widen. The analytical results are derived by estimating expected homophily from number of edges and they are in excellent agreement with the numerical results.

IV. ADJUSTING THE ALGORITHMIC RANKING

The following analytical derivation determines the fraction of minority nodes in top $d\%$ degree ranks.

The exact distribution of degrees for minority and majority is given by [2]:

$$p_i(k) = 2m^{1/\beta_i} k^{-\gamma_i} \tag{10}$$

in which $\beta = \frac{1}{\gamma - 1}$. Once normalized it gives:

$$p_i(k) = \frac{2m^{1/\beta_i} k^{-\gamma_i}}{\gamma_i - 1} \tag{11}$$

Therefore, the probability of having a node with a degree $k \geq K$ is given by:

$$\begin{aligned} p_i(k \geq K) &= \int_K^{+\infty} p_i(k) dk \\ &= \frac{2m^{1/\beta_i} K^{1-\gamma_i}}{(\gamma_i - 1)^2} \end{aligned} \tag{12}$$

Thus, if there are N_i nodes of each category in the whole network, the number of nodes with a degree $k \geq K$ is given by:

$$n_i(K) = 2N_i m^{1/\beta_i} \frac{K^{1-\gamma_i}}{(\gamma_i - 1)^2} \tag{13}$$

and the total number of nodes with a degree $k \geq K$ is then:

$$n(K) = 2m^{1/\beta_a} N_a \frac{K^{1-\gamma_a}}{(\gamma_a - 1)^2} + 2m^{1/\beta_b} N_b \frac{K^{1-\gamma_b}}{(\gamma_b - 1)^2} \tag{14}$$

If we are interested in the top d nodes, then there exists one K such as:

$$d = \frac{n(K)}{N} = \frac{1}{N_a + N_b} \left(2m^{1/\beta_a} N_a \frac{K^{1-\gamma_a}}{(\gamma_a - 1)^2} + 2m^{1/\beta_b} N_b \frac{K^{1-\gamma_b}}{(\gamma_b - 1)^2} \right) \tag{15}$$

This equation can be solved numerically to find this K. Then the number of nodes of each category in the top d nodes is given by eq. (13).

This calculation enables us to estimate the trend in Figure 3B of the original paper. Consequently, by knowing the homophily and group sizes in the network, we can determine the exponent in Eq. (10) and predict the probability of minorities to appear in top $d\%$ from Eq. (15). The degree rank then can be adjusted by the ranking algorithms to make the number of nodes from each group proportional to their group size.

V. VARYING THE LOWER-LIMIT ACTIVITY OF MINORITY

Here, we derive analytical derivations for the case of two groups of minority and majority that have an unequal number of stubs when connecting to other nodes, denoted by m_a and m_b respectively.

Let $K_a(t)$ and $K_b(t)$ be the sum of the degrees of nodes from group a and b respectively. These quantities verify:

$$K_a(t) + K_b(t) = K(t) = 2(m_a f_a + m_b f_b)t = 2Mt \tag{16}$$

since the overall growth of the network follows a Barabási-Albert process. Let us denote the relative fraction of group size for each group as f_a and f_b. For $m_a = m_b = m$ we find again the simple symmetrical case derived before. The evolution of K_a and K_b is given in discrete time by:

$$\begin{cases} K_a(t+\Delta t) = K_a(t) + \left(m_a f_a \left(1 + \dfrac{h_{aa}K_a(t)}{h_{aa}K_a(t) + h_{ab}K_b(t)}\right) + m_b f_b \dfrac{h_{ba}K_a(t)}{h_{bb}K_b(t) + h_{ba}K_a(t)}\right)\Delta t \\ K_b(t+\Delta t) = K_b(t) + \left(m_b f_b \left(1 + \dfrac{h_{bb}K_b(t)}{h_{bb}K_b(t) + h_{ba}K_a(t)}\right) + m_a f_a \dfrac{h_{ab}K_b(t)}{h_{aa}K_a(t) + h_{ab}K_b(t)}\right)\Delta t \end{cases} \tag{17}$$

which in the limit $\Delta t \to 0$ gives:

$$\begin{cases} \dfrac{dK_a}{dt} = \left(m_a f_a \left(1 + \dfrac{h_{aa}K_a(t)}{h_{aa}K_a(t) + h_{ab}K_b(t)}\right) + m_b f_b \dfrac{h_{ba}K_a(t)}{h_{bb}K_b(t) + h_{ba}K_a(t)}\right) \\ \dfrac{dK_b}{dt} = \left(m_b f_b \left(1 + \dfrac{h_{bb}K_b(t)}{h_{bb}K_b(t) + h_{ba}K_a(t)}\right) + m_a f_a \dfrac{h_{ab}K_b(t)}{h_{aa}K_a(t) + h_{ab}K_b(t)}\right) \end{cases} \tag{18}$$

These equations verify that for $h_{aa} = h_{bb} = 0$ and $h_{ab} = h_{ba} = 1$ (perfectly heterophilic network) we get:

$$\begin{cases} \dfrac{dK_a}{dt} = M \\ \dfrac{dK_b}{dt} = M \end{cases} \tag{19}$$

and thus for the evolution of the degree of a single node:

$$\begin{cases} \dfrac{dk_a}{dt} = m_b f_b \dfrac{k_a}{\sum_i q_i k_i} = m_b f_b \dfrac{k_a}{K_b(t)} = \dfrac{m_b f_b}{M}\dfrac{k_a}{t} \\ \dfrac{dk_b}{dt} = m_a f_a \dfrac{k_b}{\sum_i q_i k_i} = m_a f_a \dfrac{k_b}{K_a(t)} = \dfrac{m_a f_a}{M}\dfrac{k_b}{t} \end{cases} \tag{20}$$

which gives:

$$\begin{cases} k_a \propto t^{\rho_b} \\ k_b \propto t^{\rho_a} \end{cases} \tag{21}$$

with

$$\begin{cases} \rho_a = \dfrac{m_a f_a}{M} \\ \rho_b = \dfrac{m_b f_b}{M} \end{cases} \tag{22}$$

Similarly, for $h_{aa} = h_{bb} = 1$ and $h_{ab} = h_{ba} = 0$ (perfectly homophilic network) we get:

$$\begin{cases} \dfrac{dK_a}{dt} = 2m_a f_a \\ \dfrac{dK_b}{dt} = 2m_b f_b \end{cases} \tag{23}$$

and thus for the evolution of the degree of a single node:

$$\begin{cases} \dfrac{dk_a}{dt} = m_a f_a \dfrac{k_a}{\sum_i q_i k_i} = m_a f_a \dfrac{k_a}{K_a(t)} = \dfrac{k_a}{2t} \\ \dfrac{dk_b}{dt} = m_b f_b \dfrac{k_b}{\sum_i q_i k_i} = m_b f_b \dfrac{k_b}{K_b(t)} = \dfrac{k_b}{2t} \end{cases} \tag{24}$$

which gives:

$$\begin{cases} k_a \propto t^{1/2} \\ k_b \propto t^{1/2} \end{cases} \tag{25}$$

Let's make the hypothesis that $K_a(t)$ and $K_b(t)$ are linear functions of time, so that $K_a(t) = Ct$ and $K_b(t) = (2M - C)t$ given Eq. (16). Using Eq. (18) with $f_a = f$ and $f_b = 1 - f$, we thus have:

$$\frac{dK_a}{dt} = C = \left(m_a f_a \left(1 + \frac{h_{aa}Ct}{h_{aa}Ct + h_{ab}(2M - C)t} \right) + m_b f_b \frac{h_{ba}Ct}{h_{bb}(2M - C)t + h_{ba}Ct} \right) \tag{26}$$

So:

$$C = \left(m_a f_a \left(1 + \frac{h_{aa}C}{h_{aa}C + h_{ab}(2M - C)} \right) + m_b f_b \frac{h_{ba}C}{h_{bb}(2M - C) + h_{ba}C} \right) \tag{27}$$

This equation for C is a polynomial of order 3 and can be numerically solved. We can then derive the evolution of the degree of a single node for both groups in the general case. Let's define:

$$\begin{aligned} Y_a(t) &= h_{aa}K_a(t) + h_{ab}K_b(t) \\ &= h_{aa}Ct + h_{ab}(2M - C)t \\ &= t(h_{aa}C + h_{ab}(2M - C)) \end{aligned} \tag{28}$$

and

$$\begin{aligned} Y_b(t) &= h_{ba}K_a(t) + h_{bb}K_b(t) \\ &= h_{ba}C + h_{bb}(2M - C)t \\ &= t(h_{ba}C + h_{bb}(2M - C)) \end{aligned} \tag{29}$$

For group a, we have:

$$\begin{aligned} \frac{dk_a}{dt} &= m_a f_a \frac{h_{aa}k_a}{Y_a} + m_b f_b \frac{h_{ba}k_a}{Y_b} \\ &= \frac{k_a}{t} \left(\frac{m_a f_a h_{aa}}{h_{aa}C + h_{ab}(2M - C)} + \frac{m_b f_b h_{ba}}{h_{ba}C + h_{bb}(2M - C)} \right) \\ &= \frac{k_a}{t} \beta_a \end{aligned} \tag{30}$$

and thus:

$$k_a(t) \propto t^{\beta_a} \tag{31}$$

Similarly, for group b we have:

$$\begin{aligned} \frac{dk_b}{dt} &= m_b f_b \frac{h_{bb}k_b}{Y_b} + m_a f_a \frac{h_{ab}k_b}{Y_a} \\ &= \frac{k_b}{t} \left(\frac{m_b f_b h_{bb}}{h_{ba}C + h_{bb}(2M - C)} + \frac{m_a f_a h_{ab}}{h_{aa}C + h_{ab}(2M - C)} \right) \\ &= \frac{k_b}{t} \beta_b \end{aligned} \tag{32}$$

and thus:

$$k_b(t) \propto t^{\beta_b} \tag{33}$$

With:

$$\begin{aligned}\beta_a &= \frac{m_a f_a h_{aa}}{h_{aa}C + h_{ab}(2M - C)} + \frac{m_b f_b h_{ba}}{h_{ba}C + h_{bb}(2M - C)} \\ \beta_b &= \frac{m_b f_b h_{bb}}{h_{ba}C + h_{bb}(2M - C)} + \frac{m_a f_a h_{ab}}{h_{aa}C + h_{ab}(2M - C)}\end{aligned} \tag{34}$$

and:

$$M = m_a f_a + m_b f_b \tag{35}$$

This derivation gives an insight on how the minority can improve their overall degree growth (expressed in β) by (i) increasing their lower-limit of activity m_a and (ii) increasing the asymmetric homophily ($h_{aa} > h_{bb}$).

---

[1] Dblp. `http://dblp.uni-trier.de/`. Accessed: 2016-09-30.
[2] Réka Albert and Albert-László Barabási. Statistical mechanics of complex networks. *Reviews of modern physics*, 74(1):47, 2002.
[3] Jeff Alstott, Ed Bullmore, and Dietmar Plenz. powerlaw: a python package for analysis of heavy-tailed distributions. *PloS one*, 9(1):e85777, 2014.
[4] Aaron Clauset, Cosma Rohilla Shalizi, and Mark EJ Newman. Power-law distributions in empirical data. *SIAM review*, 51(4):661–703, 2009.
[5] Petter Holme, Christofer R Edling, and Fredrik Liljeros. Structure and time-evolution of the internet community pussokram. com. *arXiv preprint cond-mat/0210514*, 2002.
[6] Petter Holme, Fredrik Liljeros, Christofer R Edling, and Beom Jun Kim. Network bipartivity. *Physical Review E*, 68(5):056107, 2003.
[7] Mohsen Jadidi, Fariba Karimi, and Claudia Wagner. Gender disparities in science? dropout, productivity, collaborations and success of male and female computer scientists. *arXiv preprint arXiv:1704.05801*, 2017.
[8] Fariba Karimi, Claudia Wagner, Florian Lemmerich, Mohsen Jadidi, and Markus Strohmaier. Inferring gender from names on the web: A comparative evaluation of gender detection methods. In *Proceedings of the 25th International Conference Companion on World Wide Web*, pages 53–54. International World Wide Web Conferences Steering Committee, 2016.
[9] Mark EJ Newman. The structure and function of complex networks. *SIAM review*, 45(2):167–256, 2003.
[10] Luis EC Rocha, Fredrik Liljeros, and Petter Holme. Simulated epidemics in an empirical spatiotemporal network of 50,185 sexual contacts. *PLoS Comput Biol*, 7(3):e1001109, 2011.
[11] Michael PH Stumpf and Mason A Porter. Critical truths about power laws. *Science*, 335(6069):665–666, 2012.
